# Big data and deep data in scanning and electron microscopies: deriving functionality from multidimensional data sets

**DOI:** 10.1186/s40679-015-0006-6

**Published:** 2015-05-13

**Authors:** Alex Belianinov, Rama Vasudevan, Evgheni Strelcov, Chad Steed, Sang Mo Yang, Alexander Tselev, Stephen Jesse, Michael Biegalski, Galen Shipman, Christopher Symons, Albina Borisevich, Rick Archibald, Sergei Kalinin

**Affiliations:** 1 Institute for Functional Imaging of Materials, Oak Ridge National Laboratory, Oak Ridge, TN 37831 USA; 2 The Center for Nanophase Materials Sciences, Oak Ridge National Laboratory, Oak Ridge, TN 37831 USA; 3 Materials Sciences and Technology Division, Oak Ridge National Laboratory, Oak Ridge, TN 37831 USA; 4 Center for Correlated Electron Systems, Institute for Basic Science (IBS), Seoul, 151-747 South Korea; 5 Department of Physics and Astronomy, Seoul National University, Seoul, 151-747 South Korea; 6 Computer Science and Mathematics Division, Oak Ridge National Laboratory, Oak Ridge, TN 37831 USA; 7 Computational Sciences and Engineering Division, Oak Ridge National Laboratory, Oak Ridge, TN 37831 USA; 8 Computer, Computational, and Statistical Sciences, Los Alamos National Laboratory, Los Alamos, NM 87545 USA

**Keywords:** Scanning probe microscopy, Multivariate statistical analysis, High-performance computing

## Abstract

The development of electron and scanning probe microscopies in the second half of the twentieth century has produced spectacular images of the internal structure and composition of matter with nanometer, molecular, and atomic resolution. Largely, this progress was enabled by computer-assisted methods of microscope operation, data acquisition, and analysis. Advances in imaging technology in the beginning of the twenty-first century have opened the proverbial floodgates on the availability of high-veracity information on structure and functionality. From the hardware perspective, high-resolution imaging methods now routinely resolve atomic positions with approximately picometer precision, allowing for quantitative measurements of individual bond lengths and angles. Similarly, functional imaging often leads to multidimensional data sets containing partial or full information on properties of interest, acquired as a function of multiple parameters (time, temperature, or other external stimuli). Here, we review several recent applications of the big and deep data analysis methods to visualize, compress, and translate this multidimensional structural and functional data into physically and chemically relevant information.

## Review

### Introduction

The ultimate goal for local imaging and spectroscopy techniques is to measure and correlate structure-property relationships with functionality - by evaluating chemical, electronic, optical, and phonon properties of individual atomic and nanometer-sized structural elements [1]. If available directly, the information of the structure-property correlations at the single molecule, bond, or defect levels enables theoretical models to accurately guide materials scientists and engineers to optimally use materials at any length scale, as well as allow for the direct verification of fundamental and phenomenological physical models and direct extraction of the associated parameters.

Particularly significant challenges are offered by spatially inhomogeneous, partially ordered, and disordered systems, ranging from spin glasses [2,3] and ferroelectric relaxors [4,5], to solid-electrolyte interface (SEI) layers in batteries [6] and amorphized layers in fuel cells[7,8], to organic and biological materials. These systems offer a triple challenge: defining relevant local chemical and physical descriptors, probing their spatial distribution, and exploring their evolution in dynamic temperature, light, and chemical and electrochemical reaction processes. While complex, recent progress in information and application [9] of statistics suggest that such descriptions are possible; the challenge is to visualize and explore the data in ways that allow decoupling of various local dynamics under external physical and chemical stimuli.

Ideally, complete studies have to be performed as a function of global stimuli, such as temperature or uniform electric field applied to the system, as well as local stimuli, using localized electric [10-13], thermal [14-18], or stress fields [19-23] exerted by a scanning probe microscopy (an SPM) probe [24-26], either within the classical SPM platforms or combined SPM-scanning transmission electron microscopy (STEM) set-ups [27,28]. Further complications of the detection scheme in force-based SPMs require probing of the response in a frequency band around resonance (since resonant frequency can be position dependent and single-frequency methods fail to capture these changes) [29-32].

Additionally, the instrument hardware challenge is exacerbated by a wealth of extracted information at both global and local scales necessitating a drastic improvement in capability to collect and analyze multidimensional data sets. For example, probing a local transformation requires sweeping a local stimulus (tip bias or temperature) while measuring the response. Note that all first-order phase transitions are hysteretic and often slow, constraining the measurement of the kinetic hysteresis (and differentiating it from thermodynamics) by measuring the system response as a function of time. This caveat requires first-order reversal curve-type studies, which effectively increase dimensionality of the data (e.g., probing Preisach densities [33,34]).

**Figure 1:**
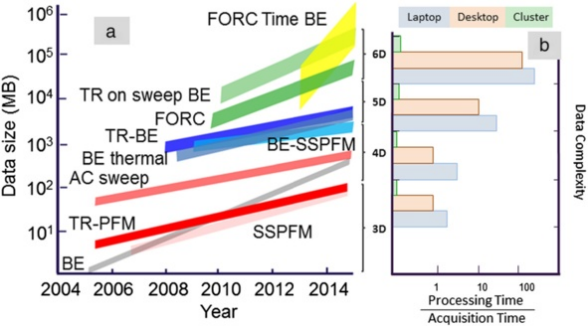
Data set size and computational power evolution. **(a)** Evolution of multidimensional data sets and their sizes over the last decade. Acronym list: BE, band excitation; SSPFM, switching spectroscopy piezoresponse force microscopy; TR PFM, time resolved piezoresponse force microscopy; BE SSPFM, band excitation piezoresponse force microscopy; TR BE, time resolved band excitation; FORC, first-order reversal curve. **(b)** Typical processing/acquisition time (smaller value is better) on a laptop, desktop, and cluster for multidimensional data sets. Hardware configurations were assumed as follows: laptop - 4-core processor, 8 GB of RAM, integrated video, and 1 hard drive approximately 1 TB of space; desktop - 12-core processor, 32 GB RAM, dedicated video, 2 hard drives, 4 TB of space; cluster - 10 nodes, each node with 8 processors at 8 cores, 20 GB of RAM, 160 GB storage space.

The arguments presented above can be summarized that in order to achieve complete probing of local transformations in SPM, 6D (space × frequency × (stimulus × stimulus) × time) data detection schemes are necessary. Figure 1a illustrates the data set size and Figure 1b the computational power evolution for 3D to 6D data sets for SPM techniques developed over the last decade. Some further details pertaining to these techniques are illustrated in Table 1. The authors also note the obvious information technology challenges associated with acquisition of large, compound data sets bring, namely, data storage, dimensionality reduction, visualization, and interpretation.

**Table 1:** **Development of multidimensional SPM methods at Oak Ridge National Laboratory**

**Technique**	**Dimensionality**	**Current data set**	**File volume**	**References**
1. Band excitation (BE)	3D, space, and *ω*	(256 × 256) × 64	32 MB	[35,68,69,127-129]
2. Switching spectroscopy PFM	3D, space, and voltage	(64 × 64) × 128	4 MB	[61,130-139]
3. Time relaxation PFM	3D, space, and time	(64 × 64) × 128	4 MB	[71,140,141]
4. AC sweeps	4D, space, *ω*, voltage	(64 × 64) × 64 × 256	512 MB	[142,143]
5. BE SSPFM	4D, space, *ω*, voltage	(64 × 64) × 64 × 128	256 MB	[11,72,144-148]
6. BE thermal	4D, space, *ω*, temp	(64 × 64) × 64 × 256	512 MB	[16,17,149,150]
7. Time relaxation BE	4D, space, *ω*, time	(64 × 64) × 64 × 64	4 MB	[151] [151-153]
8. First-order reversal curves	5D, space, *ω*, voltage, voltage	(64 × 64) × 64 × 64 × 16	2 GB	[153-157]
9. Time relaxation on sweep, BE	5D, space, *ω*, voltage, time	(64 × 64) × 64 × 64 × 64	8 GB	[158,159]
10. FORC time BE	6D, space, *ω*, voltage, voltage, time	(64 × 64) × 64 × 64 × 16 × 64	128 GB	Not yet realized

*ω*, frequency; BE, band excitation; PFM, piezoresponse force microscopy; AC, alternating current; SS PFM, switching spectroscopy piezoresponse force microscopy; FORC, first-order reversal curve.

Authors note that additional registration-based problems emerge in combined structural and functional imaging, when the information obtained via a high-resolution structural channel (imaging) is complemented by lower resolution spectroscopic probing collected on a coarse grid. These types of experiments bring about problems associated with drift correction and spatial registration of disparate data sets. Therefore, to identify relevant physical behaviors in the intrinsically high-dimensional nature of resulting data, without a deterministic physical model, clustering and unsupervised learning techniques can be utilized to establish statistically significant correlations in data sets.

As instrumental platforms and data acquisition electronics are becoming ubiquitous, efficiently storing and handling the large data sets they generate become critical. Hence, the key missing element is mastering ‘the big data’ implicitly present in the (S)TEM/SPM data sets. Here, we review some of the recent advances in the application of big data analysis techniques in structural and functional imaging data. These techniques include unsupervised learning and clustering techniques, supervised neural network-based classification, and deep data analysis of physically relevant multivariate statistics data.

### Multivariate statistical methods

The purpose of this section is to familiarize the reader with the basic unsupervised and supervised learning methods used to reduce dimensionality and visualize data behavior in a high-dimensional data set. The material presented in this section gives but a brief overview, and the reader is encouraged to explore the methods further if they have any interest in utilizing them. Minimal mathematical formalism is presented, as the focus is to explain the functional aspect of each of the methods as they are applied to spectral and imaging data, method’s strength and weakness, and give a brief overview of the input and output parameters, if any, to ease the transition to actual utility. All of the methods presented below share the same 2D data structure at the input, with rows as observations and columns as variables. This arrangement implies that in a high-dimensional data set, certain dimensions have to be combined. In our work, presented below, we combine dimensions by type, that spatial dimensions in the *X*, *Y*, or *Z* can be mixed, or similarly energy dimensions, such as AC or DC voltage. Other mixing schemes are also possible and in some areas perhaps necessary. More details are given in each of the technical sections as to how each of the methods described below was implemented.

#### Principal component analysis

Perhaps the easiest way to visualize a multidimensional data set is through principal component analysis (PCA), an approach previously reported for various applications in electron and force-based scanning probe microscopy data [35-41]. PCA has been widely used by a number of scientific fields and owes its popularity to the ease of use and wide availability of the source code in practically any programming language. The algorithm does not take any parameters besides the data itself and outputs three important results: eigenvectors (arranged from most to least information dense), the respective loading (or score) maps associated with each eigenvector, and a Scree plot that represents the information content as a function of eigenvector number. These three results allow the user to visualize the principal behaviors in the data, through eigenvectors and their loading maps, as well as judge the information content of each eigenvector via the Scree plot. PCA, however, suffers from difficulty of interpretation of higher eigenvectors, where the information content typically decreases, the qualitative nature of information content assignment, and processing speed setbacks for truly large data sets (hundreds of thousands of observations with hundreds of thousands long arrays of variables).

Here we describe the PCA functionality as it applies to a spectral data set collected on a grid. In PCA, a spectroscopic data set that is *N* × *M* pixels formed by spectra containing *P* points is converted into a linear superposition of orthogonal, linearly uncorrelated eigenvectors *w*_*k*_:

1 $$ {A}_i\left({U}_j\right)={a}_{ik}{w}_k\left({U}_j\right) $$

where *a*_*ik*_ ≡ *a*_*k*_(*x*, *y*) are position-dependent expansion coefficients or component weights, *A*_*i*_(*U*_*j*_) ≡ *A*(*x*, *y*, *U*_*j*_) is the spectral information at a selected pixel, and *U*_*j*_ are the discrete bias values at which current is measured. The eigenvectors *w*_*k*_(*U*) and the corresponding eigenvalues *λ*_*k*_ are found from the singular value decomposition of covariance matrix, **C** = **AA**^*T*^, where **A** is the matrix of all experimental data points **A**_*ij*_, i.e., the rows of **A** correspond to individual grid points (*i* = 1,.., *N*⋅*M*), and columns correspond to voltage points, *j* = 1,.., *P*. The eigenvectors *w*_*k*_(*U*_*j*_) are orthogonal and are arranged such that corresponding eigenvalues are placed in descending order, *λ*_1_ > *λ*_2_ > .... by variance. In other words, the first eigenvector *w*_1_(*U*_*j*_) contains the most information within the spectral image data, the second contains the most common response after the subtraction of variance from the first one, and so on. In this manner, the first *0*-*P* maps, *a*_*k*_(*x*, *y*), contain the majority of information within the data set, while the remaining *P*-*p* sets are dominated by noise. The number of significant components, *p*, can be chosen based on the overall shape of *λ*_*k*(*i*)_ dependence or from correlation analysis of loading maps, which correspond to each of the eigenvectors, *a*_*ik*_ ≡ *a*_*k*_(*x*, *y*). Additionally, Scree plot is used to correlate variance in each component as a function of the component’s number.

#### Independent component analysis

Independent component analysis (ICA) is a method designed to extract presumably independent signals mixed within the data. Much like PCA, the output is a collection of independent spectra and their loading maps. Unlike PCA, however, the order of ICA components is insignificant, and ICA takes in some input parameters and generally takes longer to run than PCA. One of the key ICA parameters is the number of independent components, a decision that can be highly non-trivial to make. Another often overlooked parameter is the number of principal components to retain; ICA uses PCA as a filter, and for low-dimensional data sets, or data sets with relatively few observations, the last retained principal component plays a huge role in the quality of the signal separation, as it may allow or bar certain details in your data to be presented to the algorithm.

ICA is part of a family of algorithms aimed at blind source separation, where the objective is to ‘un-mix’ several sources that are present in a mixed signal [42]. The data variables are assumed to be linear mixtures of some unknown latent variables, and the mixing system is also unknown. ICA assumes that the latent variables will be non-Gaussian and therefore mutually independent. The problem of blind source separation can be modeled in the following manner:

2 $$ x=As $$

where ***s*** is a two-dimensional vector containing the independent signals, *A* is the mixing matrix, and *x* is the observed output. As the initial step, ICA whitens the data to remove any correlation; in other words, we are after a linear transformation **V** such that if

3 $$ y=\boldsymbol{V}x $$

We would like to find the identity **I** by

4 $$ E\left\{yy\hbox{'}\right\}=\mathbf{I} $$

This is possible by **V** = **C**^−1/2^ where **C** = E{xx′} giving us

5 $$ E\left\{yy\hbox{'}\right\}=E\left\{\boldsymbol{V}xx\hbox{'}\boldsymbol{V}\hbox{'}\right\}={\boldsymbol{C}}^{-1/2}\boldsymbol{C}{\boldsymbol{C}}^{-1/2}=\mathbf{I} $$

After the whitening, independent signals can be approximated by the orthogonal transformation of the whitened signal by rotating the joint density of the mixed signals in a way to maximize the non-normality of the marginal densities.

#### Bayesian de-mixing

Bayesian de-mixing is a very powerful technique that shines where PCA and ICA fall short. First and foremost, Bayesian de-mixing returns a quantitative result with the units of de-mixed spectra being the units of the input data.

The de-mixed vectors are also always positive and sum to one, which makes the transition from statistics to science quite natural. There are many optional parameters that can be tweaked within the Bayesian code, but typically at least the number of independent components is required. The disadvantage of the Bayesian method is speed, and additional insight is necessary to optimize the algorithm. Typically, in our analysis flow, we start with PCA and ICA to identify the parameter space; once the region of interesting solutions or phenomena is identified, we perform Bayesian de-mixing.

While a plethora of Bayesian-based statistics methods exist, we have found the algorithm provided by Dobigeon et al. to be the fastest and easiest to use [43]. The Bayesian approach assumes data in a **Y = MA + N** form, where observations **Y** are a linear combination of position-independent endmembers, **M**, each weighted with respective relative abundances, **A**, and corrupted by an additive Gaussian noise **N**. This approach features the following: the endmembers and the abundance coefficients are non-negative, fully additive, and sum-to-one [44-47].

The algorithm operates by estimating the initial projection of endmembers in a reduced subspace via the N-FINDR [48] algorithm that finds a simplex of the maximum volume that can be inscribed within the hyperspectral data set using a non-linear inversion. The endmember abundance priors along with noise variance priors are picked from a multivariate Gaussian distribution found within the data, whereas the posterior distribution is based on endmember independence calculated by Markov Chain Monte Carlo, with asymptotically distributed samples probed by the Gibbs sampling strategy. An additional, unique aspect of Bayesian analysis is that the endmember spectra and abundances are estimated jointly, in a single step, unlike multiple least square regression methods where initial spectra should be known [43].

#### Clustering

A very natural way to analyze data is to cluster it. There are many algorithms available that have a variety of built-in assumptions about the data and as such could predict the optimal clustering value, order clusters based on variance, or other distance metrics, etc. We present a method, *k*-means clustering, which is rather flexible and easy to find on a variety of platforms and in many programming languages. The only required input value for *k*-means is the number of clusters; however, additional variables such as the distance metric, number of iterations, how the initial sample is calculated, and how to handle unorthodox data events can all have drastic effects on the results. This clustering algorithm is moderately fast and returns a simple index of integers which enumerates each observation to its respective cluster. The biggest downside of *k*-means clustering algorithm is the random cluster ordering on the output; however, this information can be indirectly accessed by looking at the average distance between clusters (based on the supplied metric) as well as the number of points in the cluster.

*K*-means algorithm divides *M* points in *N* dimensions into *K* clusters so that the within-cluster sum of squares

6 $$ \arg \min {\displaystyle \sum_{i=1}^k}{\displaystyle \sum_{x_j\in {S}_i}}\left|\right|{x}_j-{\mu}_i\left|\right|{}^2, $$

where ***μ***_***i***_ is the mean of points in *S*_*i*_, is minimized [49,50]. Here, we have used an implementation of the *k*-means algorithm that minimizes the sum, over all clusters, of the within-cluster sums of point-to-cluster-centroid distances. As a measure of distance (minimization parameter), in our data, we have typically used sum of absolute differences with each centroid being the component-wise median of the points in a given cluster.

#### Neural networks

Artificial neural networks (ANNs) are an entire family of algorithms, modeled after the neural system found in the animal kingdom, used to estimate unknown functions that may have a very large number of inputs. ANNs are similar to the biological neural system in that they perform functions collectively and in parallel by the computational units, as opposed to have each unit a clearly assigned task. In a mathematical sense, neuron’s function *f*(*x*) can be defined as a mixture of other function *g*(*x*) with weighting factors *w*_*i*_ where *g*(*x*) is a non-linear weighted sum of *f*(*x*)

7 $$ f(x)=K\left({\displaystyle \sum_i}{w}_ig(x)\right) $$

Here *K* is commonly referred to as an activation function that defines the node output based on the set of inputs.

What has attracted people to ANNs is the possibility of those algorithms to simulate *learning*. Here by *learning* we imply that for a specific task and a class of functions, there is a set of observations to find that that relates the solutions of the set of functions. To utilize this concept, we must imply a cost function *C* which is a measure of how far away a particular solution is from the optimal solution. Consider the problem of finding a model ***f*** which minimizes the cost function

8 $$ C=E{\left[\left(f(x)+y\right)\right]}^2 $$

for some set of points (*x*, *y*) from a distribution **D**. In such a case, the finite number of samples **N** from **D** would minimize the cost function as

$$ C=\frac{1}{N}{\displaystyle \sum_{i=1}^N}E{\left[\left(f\left({x}_i\right)+{y}_i\right)\right]}^2 $$

As the reader may note, ultimately, the cost function is dictated by the problem we are trying to solve. In the case of an unsupervised learning problem, we are dealing with a general estimation problem, so the cost function is chosen to reflect our imposed model on the system. In the case of supervised learning, we are given a set of examples and to aim to infer the mapping based on the data in the training and other data sets. In the simplest case scenario, the cost function would be a mean-squared error type, which would try to minimize the average error between the network’s output and the target values of the example sets.

### Spectral domains

We illustrate the applications of multivariate data analytics techniques to multidimensional functional spectroscopies, which include bias, current, frequency, and time channels in SPM and electron energy loss spectroscopy (EELS) in STEM. The analysis involves signal decomposition along the energy or stimulus direction, whereas the spatial portion of the signal is left pristine. In this section, we illustrate analysis via unsupervised and supervised learning algorithms for scanning tunneling spectroscopy (STM) and atomic force microscopy (AFM)-based electromechanical force spectroscopies.

#### 3D data - CITS in STEM

In STM, an electrically conductive tip is brought into a current tunneling distance to a conductive sample [51,52]. In *Z*-imaging mode, the tip is scanned over the sample and a *Z* feedback is used to maintain a constant current while simultaneously adjusting and collecting the position of the feedback. Conversely, in the current imaging mode, *Z* height is kept constant and the current variation is measured [53]. In current imaging tunneling spectroscopy (CITS), the measurement is performed at an individual spatial point located at an (*x*, *y*) position on a grid with the current *I* recorded for a given applied voltage waveform *U*. The final data object is a 3D stack of spectral current images *I*(*x*, *y*, *U*), where *I* is the detected current, *U* is the tip bias, and (*x*, *y*) are spatial surface coordinates of the measurement [54].

**Figure 2:**
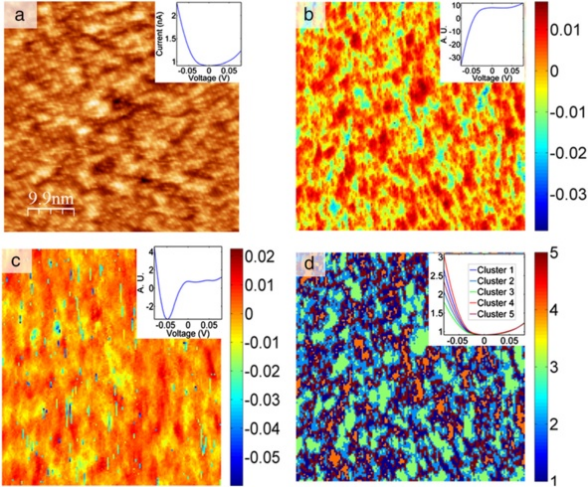
Unsupervised learning methods, PCA, and *k*-means on the FeSeTe CITS data. **(a)** 150 × 150 pixel CITS data from the Z-channel before the feedback is disabled for the IV spectroscopy. The inset shows the average IV for the data set. **(b)** Second PCA loading of the CITS data shown in (a); the inset shows the eigenvector. **(c)** Third PCA loading of the CITS data shown in (a); the inset shows the eigenvector. **(d)** *k*-means clustering results for a five-cluster case; the inset shows the mean IV for each cluster.

In this example of an Fe-based superconductor FeTe_0.55_Se_0.45_ (*T*_*c*_ = 15 K), CITS imaging was performed on a 150 × 150 grid at −0.05 to 0.05 V bias, sampled over 256 points. The layered FeTe_0.55_Se_0.45_ compound is a prototypical layered, high-temperature superconductor, described at some length in prior publications [55,56]. These data were collected on a 50 × 50 nm^2^ area, i.e., each pixel corresponds to 1 × 1 Å^2^; the lattice constant of the material is 3.8 Å. The Z position of the piezo was recorded on a separate channel prior to the *Z*-feedback disengage at the beginning of each bias waveform sequence, resulting in a 150 × 150 pixel topographic map. The *Z* channel spectroscopy image and the average spectroscopy signal are shown in Figure 2a, and the inset in the top right corner is the average current-voltage (IV) curve for the entire image. Approximate acquisition time for the CITS map was 8 to 10 h which resulted in some drift, apparent in the bottom portion of Figure 2a.

The spatial variability of the electronic behavior across the surface was analyzed using PCA [35-37,39,57]. PCA eigenvector and loading map pairs for components 2 and 3 are shown in Figure 2b,c for the FeTe_0.55_Se_0.45_ CITS data set. It is useful to analyze the eigenvectors and loadings simultaneously to examine the changes in the signal first (the eigenvector here) and its spatial distribution next (the loading). From a statistical perspective, we are mapping sources of electronic inhomogeneity arising from the negative portion of the IV curve in both components, as illustrated in Figure 2b,c. The second eigenvector shown in Figure 2b (upper right corner inset) shows an increase in the −0.05 to 0 V half of the range, where the average signal has a negative slope in the same region. In the third eigenvector, loading pair shown in Figure 2c, the variation is also more prominent in the negative half of the bias range where the current forms a well, compared to the smooth decay behavior in the average IV. Therefore, changes in the current at negative bias are strong sources of data variance in the system and can be attributed to chemical segregation at the surface.

While the components are statistically significant and reflect major changes in the variability of the data, the connection to the physical properties PCA highlights is always non-trivial. This is mostly due to the fact that information variance, the property with respect to which PCA organizes the data, is sensitive to the variability in the signal, rather than the physical origin of the change. This suggests that PCA allows one to de-noise, de-correlate, and visualize spatial variability of the response but does not directly yield additional knowledge with respect to the effects that are being studied. In the case of CITS data on FeTe_0.55_Se_0.45_, results of the third component, Figure 2c, can be legitimately questioned as the loading map seems to suggest behavior that is erratic and typical of an unstable tip surface tunneling regime. It is then necessary to use other methods in order to supplement PCA results and determine the underlying source of variance in the signal and its relevance to the problem at hand, as will be illustrated by Bayesian de-mixing analysis of the local conductance behavior in the section ‘Deep learning’ [40,58,59].

Another commonly used unsupervised learning method that reflects major organization in the data structure is *k*-means clustering. Insight into the spatial variability of the electronic structure on the surface inaccessible by PCA can be gained from the clustering analysis of the CITS data [60], by *k*-means clustering. As a measure of distance (minimization parameter), we have used the sum of absolute differences with each centroid being the component-wise median of the points in a given cluster.

The *k*-means result for five clusters using the square Euclidean distance metric is shown in Figure 2d, with the inset in the top right showing the mean IV curves for the individual clusters (color-coded respectively). As seen in the *k*-means clustering result, the mapping is indeed sensitive to the changes in the negative bias portion of the IV curve. Here we see clustering that is based on variance of conductivity or alternatively the width of the band gap. Perhaps a more interesting observation is the spatial distribution of the clusters, where the regions of the highest maximum current (cherry red) and lowest maximum current (green) are segregated and in most cases surrounded by patches of varying conductivity. Note that in this result, single pixel and short line like agglomerates of pixel outliers seen in Figure 2c are absent. Overall, the behavior is more in line with the results of the second PCA component.

#### 4D and 5D data - band excitation spectroscopy analysis

The multivariate analysis of a higher-dimensional data set (beyond the 3D) is effectively illustrated by a band excitation piezoresponse force spectroscopy (BEPS) data set. This technique probes the electromechanical response of materials, which is directly related to the material’s ferroelectric state. The spectroscopic version of piezoresponse force microscopy (PFM) probes the local ferroelectric switching induced by the DC bias applied to the tip via a dynamic electromechanical response, effectively yielding the local piezoresponse loop.

**Figure 3:**
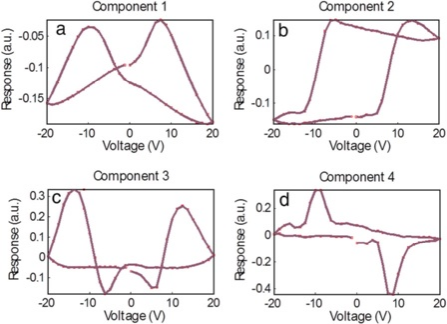
PCA of band excitation piezoresponse force spectroscopy (BEPS) data. **(a)** First eigenvector (principal component) of the BEPS data. **(b)** Second eigenvector (principal component) of the BEPS data. **(c)** Third eigenvector (principal component) of the BEPS data. **(d)** Fourth eigenvector (principal component) of the BEPS data.

**Figure 4:**
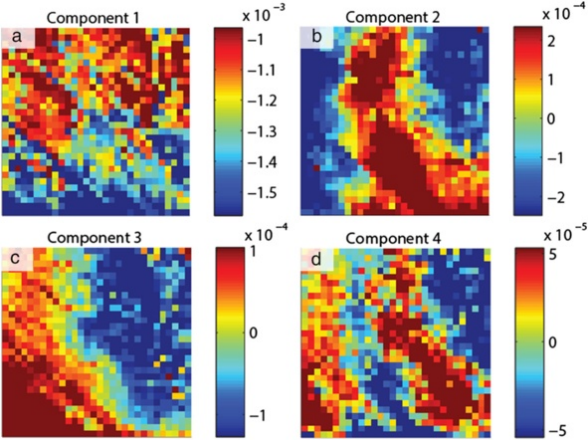
PCA loading maps of band excitation piezoresponse force spectroscopy (BEPS) data. **(a)** First loading map associated with the principal components in Figure 3 of the BEPS data. **(b)** Second loading map associated with the principal components in Figure 3 of the BEPS data. **(c)** Third loading map associated with the principal components in Figure 3 of the BEPS data. **(d)** Fourth loading map associated with the principal components in Figure 3 of the BEPS data.

The data shown in this section consist of a 30 × 30 grid of points (*x*, *y*), where each point contains a ferroelectric hysteresis loop captured by applying a voltage waveform and measuring the piezoresponse [61-66]. The sample is a relaxor ferroelectric PMN-0.28PT sample, which is in the ferroelectric phase and displays strong piezoelectricity. The amplitude of the piezoelectric response *A* is then a function of (*x*, *y*, *V*), *A* = **A**(*x*, *y*, *V*); the voltage waveform consists of 64 voltage steps, implying 64 spatial maps of amplitude (one for each voltage step). Alternatively, at each (*x*, *y*) location, one can inspect the ferroelectric hysteresis loop, i.e., the amplitude as a function of voltage for *x* = *x*_1_, *y* = *y*_1_. In total, 900 hysteresis loops were captured that could be analyzed, and PCA was undertaken for this data set (method described in the previous section), with the first four eigenvectors in Figure 3a,b,c,d with their respective loading maps shown in Figure 4a,b,c,d. The first component represents the mean of the data set (since the mean captures the most variance in the data), and subsequent components detect the variations in the (amplitude) hysteresis loop shape from iteratively deviating from the mean. Note that the components can arbitrarily switch sign, but in these instances, the eigenvalues will also be reversed to preserve the correct orientation of the reconstructed data set. The second component in Figure 3b is a measure of the asymmetry of the loop (related to ferroelectric imprint), while the third appears to widen the loop (i.e., change the coercive field). Finally, the fourth component displays non-trivial features which, in the reconstructed data set, appear as mound-like features on either side of the switching cycle. The spatial maps illustrate significant heterogeneity for each component, as a result of the widely varying ferroelectric switching behavior across the sample. Thus, PCA once again provides an effective method to quickly map the trends in the data set.

Although PCA is useful in visualizing the structure of the data, there are no physically meaningful constraints on the eigenvectors. For example, if it is known (or postulated) that the measured signal is a linear combination of *n* independent signals, one may want to determine the pure components that correspond to each of these cases. For this particular problem, the ICA [42] technique provides a solution and allows de-mixing of signals into a user-defined number of vectors (components), with the constraint that the components must be statistically independent.

Consider the amplitude signal *A* in the BEPS example written as a sum of four independent components *s*_*i*_ (*i =* 1,…4), with mixing coefficients *c*_*i*_ (*i* = 1,…4), the amplitude is then described by Equation 9:

9 $$ A\left(x,\;y,\;V\right)={c}_1\left(x,\;y\right){s}_1(V)+\dots +{c}_4\left(x,\;y\right){s}_4(V) $$

**Figure 5:**
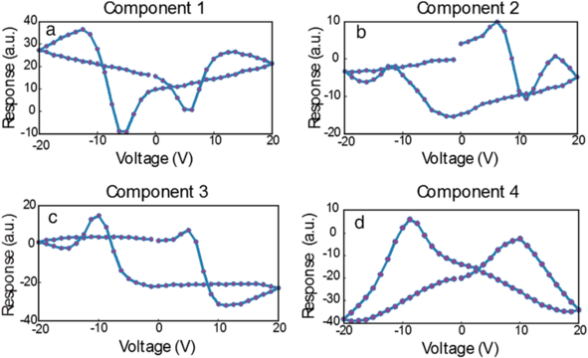
ICA of band excitation piezoresponse force spectroscopy (BEPS) data. **(a)** The first independent component from the ICA analysis of the BEPS data. **(b)** The second independent component from the ICA analysis of the BEPS data. **(c)** The third independent component from the ICA analysis of the BEPS data. **(d)** The fourth independent components from the ICA analysis of the BEPS data.

**Figure 6:**
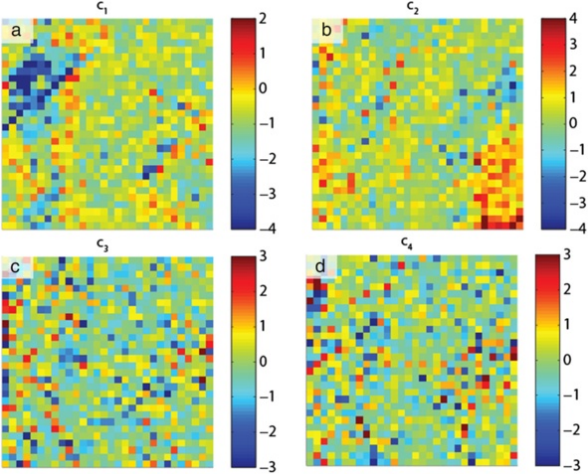
ICA of band excitation piezoresponse force spectroscopy (BEPS) data. **(a)** Mixing coefficient maps associated with the first independent components in Figure 5. **(b)** Mixing coefficient maps associated with the second independent components in Figure 5. **(c)** Mixing coefficient maps associated with the third independent components in Figure 5. **(d)** Mixing coefficient maps associated with the fourth independent components in Figure 5.

ICA can be used to find *s*_*i*_(*V*) and *c*_*i*_(*x*,*y*). In essence, such a transformation allows the data to be represented by a specific number of independent ‘processes’ (components) that are mixed in the final signal, while the coefficients determine the relative weights of each process to the total signal contribution. The results of this de-mixing are shown in Figure 5, with the independent components shown in Figure 5a,b,c,d and the corresponding mixing coefficients shown in Figure 6a,b,c,d. Unlike in PCA, there is no particular ordering to the components; however, similar to PCA, the components may flip in sign.

The spatial maps of the mixing coefficients show variability in the response and are markedly different from the PCA eigenvalue maps; for example, the bottom right area of the sample displays high response of the second component (Figure 6b), which increases the area enclosed within the left side of the butterfly loop. In this example, note that there is no reason for there to be four components to the hysteresis loop, i.e., we illustrate an example of the method, but based on component shape, there should be *at least* four as all components appear significantly different. Importantly, the fourth component displays a near-ideal ferroelectric loop (Figure 5d), and the strength of this component with respect to the other components can be seen as an indication of the degree of purely ferroelectric switching in those regions, as opposed to other components that appear to result from dominating influences by surface charges, polar nanoregions, or field-induced phase transformations. For instance, the first component appears largest in the top-left corner of the region studied (Figure 6a), and the coercive fields for this component are much lower, possibly due to the increased propensity of field-induced phase transformations (likely rhombohedral to tetragonal [67]) in this area. Thus, ICA is a highly useful method for blind source separation and provides a powerful method accompanying PCA to de-mix signals where the number of constituent components is either known from physics or can be postulated.

#### Supervised learning

**Figure 7:**
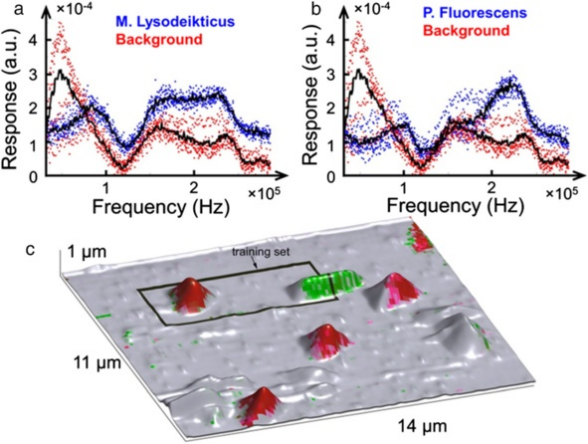
Functional recognition imagining of a bacterial sample. **(a, b)** Electromechanical response spectra of two bacterial species and background. **(c)** AFM topographic image of an area containing bacteria with the training set marked with a rectangular box; coloration indicates neural network identification, with green corresponding to *P. fluorescens*, red to *M. lysodeikticus*, and gray to the background.

Functional recognition imaging is an example of the supervised learning approach that employs artificial neural networks. The process of recognition obviates the need for sophisticated analytical models, instead relying on statistical analysis of the complex spectroscopic data sets. Nikiforov et al. [68] describe functional recognition imaging of bacterial samples containing live *Micrococcus lysodeikticus* and *Pseudomonas fluorescens* on a poly-L-lysine-coated mica substrate. These bacteria differ in shape and therefore present a good modeling system for creating training data sets. The spectroscopic data were provided by the band excitation PFM method [69,70] in the form of the electromechanical response vs. excitation frequency spectra collected across chosen sample areas that contained both of the bacteria species, the substrate and debris. The spectra (shown in Figure 7a,b) of bacteria and substrate clearly contain unique signatures allowing for their identification on the *single-pixel* (i.e., spectral) level. Note that these electromechanical responses originate in a very complex interplay of different interaction mechanisms between the AFM tip and surface: long-range electric double layer forces and bacterial electromobility and flexoelectric properties, with the control over cantilever dynamics governed by the boundary conditions at the tip-surface junction. This complexity precludes analytical modeling of the data but provides enough statistical significance for the successful application of a neural network.

The training of the neural network was performed on a region of the sample outlined with a black box in Figure 7c. The inputs to the network were the first six components of the principal component analysis decomposition (here, acting as a filter) of the data set within the training region. A network of three neurons was trained repeatedly on multiple examples until a minimal error was achieved. Following training, the network was presented with the data set collected on the whole area shown in Figure 7c and it correctly identified both of the bacterial species. Interestingly, other topographical features, distinct from the substrate, were classified as background, which identifies them as non-bacterial debris. However, a small relatively flat region (right upper corner in Figure 7c) was classified as *M. lysodeikticus*, implying that this region could be covered in a membrane of lysed bacteria of that species. Thus, supervised learning presents a powerful image recognition tool that can identify objects based on a small subset of information provided in the training set. Even though successful neural network operation requires extensive training for accuracy, the computational cost during operation is infinitesimal. The illustrated example was computed on a typical user desktop without additional high-end components or computational clusters. Similarly, neural network approaches can be extended to training on theoretical model outputs, with the experimental results presented for analysis. Examples include functional fits to relaxation parameters [71] or Ising model simulations [72,73].

#### Deep learning

In this section, we discuss the pathways to establish correspondence between statistical analysis and a physical model, i.e., to transition from a search for correlation to a search for causation. The previously introduced first-order reversal curve current-voltage (FORC-IV) SPM technique [74] has been deployed in imaging and analysis of spatially uniform Ca-substituted BiFeO_3_ and NiO systems [74,75]. Those studies have shown that the locally measured hysteresis in the FORC-IV curves is related to changes in electronic conduction sensed by the tip in response to a bias-induced electrochemical process, and the area of the IV loop is overall indicative of local ionic activity. FORC-IV spectroscopic imaging modes lack adequate data analysis and interpretation pathways due to the flexible, multidimensional nature of the data set and the volume of the data collected. In this example, we combine FORC-IV measurements with the multivariate statistical methods based on signal de-mixing, in order to discriminate between different conductivity behaviors based on the *shapes* of the IV curves in the full spectroscopic data set.

**Figure 8:**
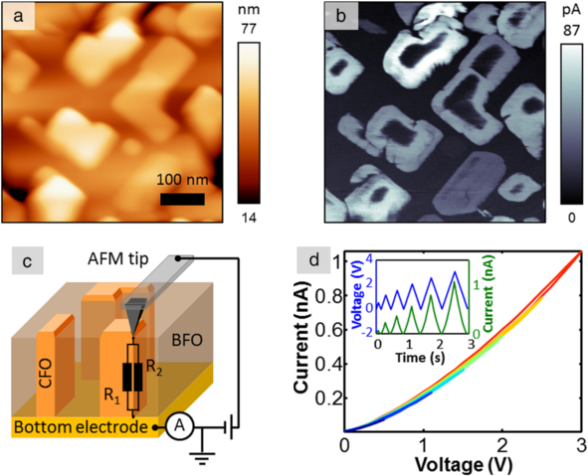
c-AFM on CFO-BFO. **(a)** Contact mode c-AFM topography of CFO-BFO. **(b)** c-AFM of CFO-BFO at 100 mV tip bias. **(c)** Schematic of the CFO-BFO nanocomposite sample and experimental setup. **(d)** FORC-IV average current for all pixels at 87% humidity; the inset shows FORC-IV bias waveform (blue) and current response (green) for the 87% humidity case.

A CoFe_2_O_4_-BiFeO_3_ nanocomposite thin film (CFO-BFO, Figure 8c) was grown by pulsed laser deposition and is a self-assembled, tubular heterostructure that forms spontaneously due to segregation of the perovskite BFO matrix and the CFO spinel inclusions [58,76]. The FORC-IV spectroscopy was performed at humidity values ranging from 0% to 87%, with an intermediate 58% case also shown. To gain insight into the fine structure of the CFO pillars and the CFO-BFO tubular interface, we first used conductive AFM (c-AFM) to image areas of size 500 × 500 nm^2^ of the film and then collect FORC-IV data using a waveform with six triangular pulses and a maximum DC peak bias of 3 V on a 50 × 50 pixel grid overlaid on the imaged area. This corresponds to a pixel size of 10 × 10 nm^2^. Figure 8a,b illustrates a typical ambient c-AFM result including topography and a conductivity map collected at 100 mV shown in Figure 8b. Notice that the current is maximized at the edges of the pillars extending into the BFO matrix. Furthermore, some of the pillars feature a central spot of low conductivity, while others are fully conductive. The inset of Figure 8d illustrates details of the FORC-IV experiment, specifically the applied triangular bias waveform, shown in blue, and average current response for the entire 50 × 50 pixel spectroscopy area as a function of time, shown in green. Figure 8d shows the average current loop for the whole bias waveform as a function of voltage; note that these curves are essentially featureless with little to no hysteresis and are highly smooth in both forward and reverse voltage sweep directions.

The multidimensional nature of these data, combined with the lack of analytical or numerical physical models, naturally calls for multivariate statistical analysis in order to extract the most comprehensive view of the physical behavior of the CFO-BFO system. While PCA and ICA are powerful methods that allow one to take a closer look into the structure of the data, a preferable method would preserve physical information in the data and allow fully quantitative analysis. Such a method will separate the data into a combination of well-defined components with clear spectroscopic behavior that has an intensity weight component, providing insight into the spatial distribution of the behavior. Ideally, these components should be physically viable, well-behaved, positive, have additive weights, etc. This level of analysis can be achieved by Bayesian linear de-mixing methods, specifically an algorithm conglomerate introduced by Dobigeon et al. [43].

The main advantage of these methods is a quantitative, interpretable result where the final endmembers are non-negative, in the units of input data, and with all of the respective abundances adding up to 1. Therefore, at each location, the data is decomposed into a linear combination of spectra where each pixel in the probed grid consists of a number of components (i.e., conducting behaviors) present in a corresponding proportion. Note that these constraints allow a direct transition from statistical analysis to physical behavior. By making the abundances additive and the endmembers positive, we can begin assigning physical behavior to the shape and nature of the endmember curves. By extension, analysis of the endmember loading maps adds the spatial component to the behavior that non-statistical methods of analysis lack entirely.

**Figure 9:**
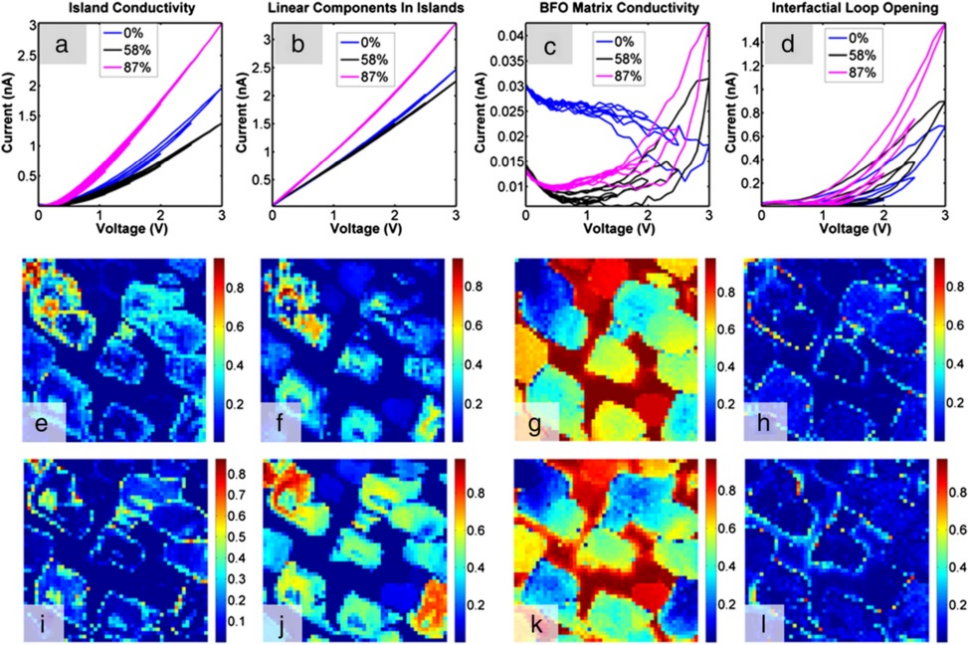
Bayesian de-mixing on CFO-BFO FORC-IV data. Top row are de-mixed vectors for the 0%, 58%, and 87% humidity; middle row are loading maps for the 87% humidity; and bottom row are loadings for the 0% humidity. **(a)** Island conductivity Bayesian de-mixed component for the 0%, 58%, and 87% humidity. **(b)** Inner island conductivity Bayesian de-mixed component for the 0%, 58%, and 87% humidity. **(c)** BFO matrix conductivity Bayesian de-mixed component for the 0%, 58%, and 87% humidity. **(d)** Interfacial conductivity between the CFO and BFO matrix Bayesian de-mixed component for the 0%, 58%, and 87% humidity. **(e)** Island conductivity loading map for the 87% humidity. **(f)** Inner island conductivity loading map for the 87% humidity. **(g)** BFO matrix conductivity loading map for the 87% humidity. **(h)** Interfacial conductivity loading map between the CFO and BFO matrix at 87% humidity. **(i)** Island conductivity loading map for the 0% humidity. **(j)** Inner island conductivity loading map for the 0% humidity. **(k)** BFO matrix conductivity loading map for the 0% humidity. **(l)** Interfacial conductivity loading map between the CFO and BFO matrix at 0% humidity.

Following the experiments at 0%, 58%, and 87% humidity, we performed Bayesian de-mixing of the current signal into four components. The reasons for choosing four components and the supporting arguments are discussed in detail by Strelcov et al. [58]. De-mixed vectors for all three experiments as well as loadings for the 0% and 87% humidity cases are shown in Figure 9. The de-mixed components correspond to 1) electronic transport through a potential barrier (Figure 9a) active in the central and outer parts of the CFO pillars, 2) an Ohmic conductance (Figure 9b) present in the bulk of CFO islands, 3) negligible conductivity of the CFO matrix (Figure 9c), and 4) interfacial electrochemistry that generates hysteresis in IV curves (Figure 9d). Evidently, although an increase in humidity level brings about an increase in overall conductivity, the response of individual components is much more complex, implying several competing mechanisms. A decrease in CFO conductivity (component 1) on humidity increase from 0% to 58% might be due to formation of water meniscus at the tip-surface junction, which effectively decreases the strength of electric field and hampers transport through the barrier. On the other hand, the ohmic component stays almost unaffected in these conditions, being dependent on potential difference between the electrodes, rather than electric field strength. A further increase in humidity to 87% not only increases maximal current in components 1 and 2, but also leads to intensity shift from component 1 to component 2 in the abundance maps (cf. Figure 9e,i and Figure 9f,j pairwise), i.e., decreasing the height of potential barrier of electron/hole injection from the tip into the nanocomposite. This might be due to generation of H^+^ ions by the tip via water electrosplitting. Finally, the fourth - electrochemical component - steadily intensifies as the humidity increases from 0% to 87%, as expected from water electrolysis. The threshold voltage decreases and the reaction zone widens, as can be observed by comparing Figure 9h and Figure 9l. This exemplifies the ability of deep data analysis not only to highlight statistically significant traits in multidimensional data, but also to extract physically and chemically relevant behaviors, preserving the units of measurement in the process.

### Image domain

The clustering and dimensionality reduction algorithms used in the previous sections are equally applicable to analysis in image coordinate space, exploring the correlation between individual structural elements found in the image itself. These can originate from both contrast and shape-based features contained in the image, as well as an analysis of features that are mathematically condensed into a representative set.

#### Sliding Fourier transform

**Figure 10:**
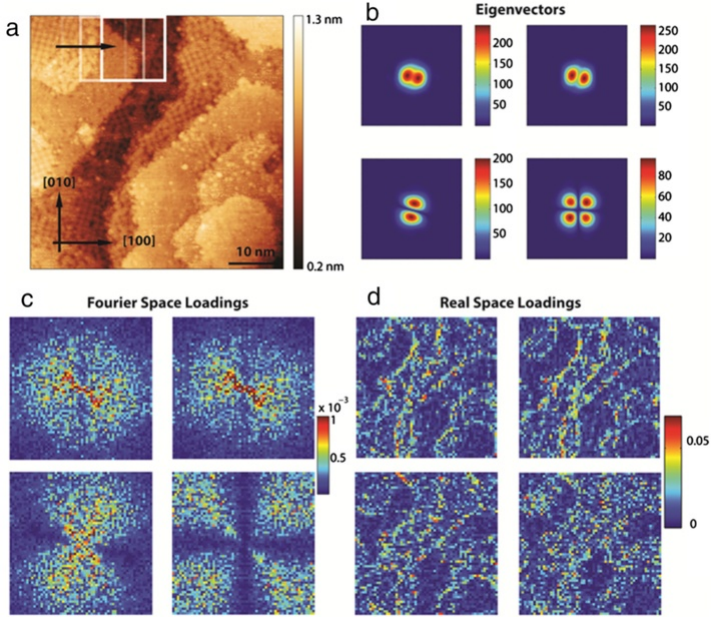
Sliding FFT on an STM image of La_5/8_Ca_3/8_MnO_3_. **(a)** STM topography image of 16-unit cell sample of La_5/8_Ca_3/8_MnO_3_ grown on (001) SrTiO_3_. Sliding FFT was carried out, which consists of creating a window (white square in image) in which the FFT is captured, and subsequently sliding the window across the image a preset distance and recording the next FFT of the windowed area until the entire surface is covered to produce the data set. PCA of this data set was performed, and the first four components are shown in **(b)**, with the respective Fourier loadings **(c)**. Transforming the loadings to real space allows investigating the spatial distribution, and the first four real-space components are shown in **(d)**.

As an example of image domain analysis, we demonstrate a sliding fast Fourier transform (FFT) filter [77] for analysis of surface reconstructions on epitaxially grown films of La_5/8_Ca_3/8_MnO_3_ (LCMO). The image analyzed in Figure 10a, is a 50 × 50 nm^2^ STM topography image (captured at a resolution of 512 × 512 pixels). We analyze a small window of the surface (outlined as a white square in the figure, of size 128 × 128 pixels) and generate a FFT image of that area. The window is then slid across the image by a preset number of pixels (in this case, 8), and the FFT image is captured once again; this process is repeated until the window has covered the entire real-space image in the horizontal direction. The window is then stepped in the *y*-direction and the process repeated until all areas of the image have been covered. The output of this procedure is a large array (here, size 60 × 60 × 128 × 128) of position-dependent FFT images, which are then analyzed using PCA to identify the trends and the spatial variations in the data set. The 2D PCA eigenvectors are plotted in Figure 10b, with the first two eigenvectors showing spacing closely aligned across the [63] direction, while the third eigenvector shows periodicity more closely aligned in the [010] direction. The loading maps for the eigenvectors, in Fourier space, are shown in Figure 10c, and the real-space loadings are plotted in Figure 10d. The real-space loadings readily identify the sites of interest, as measured by changes in the lattice (be it spacing or angle). The second real-space loading is particularly adept at finding edges of the ordered/disordered areas, as well as ordered but differently oriented lattices. The fourth component identifies the regions in the image where there is a clear lattice. These results show the promise of using the sliding FFT/PCA algorithm to quickly identify the types of periodicity and their spatial distribution in an image.

#### Clustering and classification of atomic features

**Figure 11:**
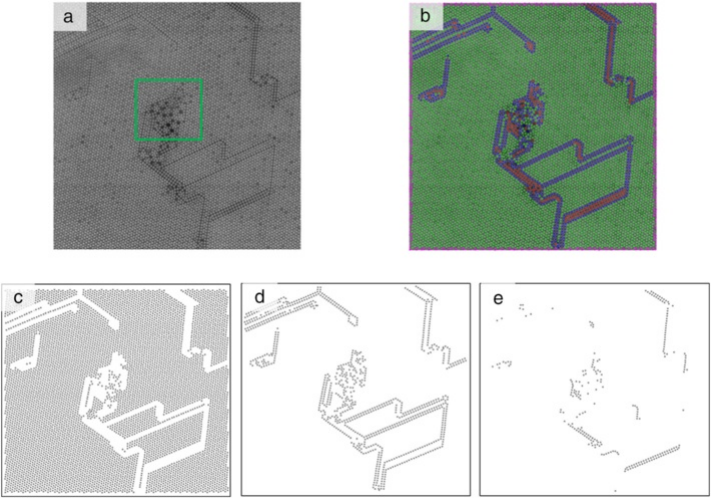
*K*-means clustering results for four clusters on the STEM-HAADF image of the Mo-V-Sb-Ta oxide two-phase catalyst. **(a)** Raw STEM image. **(b)** *K*-means result for four clusters based on the length to the six nearest neighbors distance metric. **(c)** Sole cluster 1. **(d)** Sole cluster 2. **(e)** Sole cluster 3.

An example of correlative learning is shown by the *k*-means clustering algorithm on atomically resolved scanning transmission electron microscopy (STEM) images. We demonstrate phase separation based on local analysis of the atomic neighborhood. We identify all atoms in the image, assign them to nearest neighbors (six neighbors in this case), and perform clustering analysis on the relative bond lengths of the resultant six-member array set. The material system is a Mo-V-M-O (M = Ta, Te, Sb, and Nb) mixed oxide, which is one of the most promising catalysts for propane (amm)oxidation, with improvement of their performance being widely pursued [78-80]. In this system, the catalytic performance can be altered by intermixing two phases referred to as the M1 (ICSD no. 55097) and the M2 (ICSD no. 55098) phases, with the correlative analysis serving as a quantitative framework that allows to separate M1 and M2 as well as estimate their relative contributions as a function of the catalytic conversion. Figure 11a is a scanning transmission electron microscopy high-angle annular dark-field imaging (STEM-HAADF) image of the Mo-V-Sb-Ta oxide with the M1 (highlighted by a green square) and the remaining area being largely a M2 phase speckled with M1 dislocations. Figure 11b shows the result of the *k*-means clustering for four clusters (one of the clusters consists of the edge atoms in the image and is not shown) that clearly delineate the M1 phase members (shown in red), M2 matrix phase (shown in green), and a strain relieving interface between the two shown in blue. Figure 11c,d,e shows each cluster individually. While the example is relatively simple, it serves to bridge machine learning methods with high-quality experimental data that, due to its intrinsic complexity, is typically only qualitatively analyzed. While it may be possible to manually distinguish phases and assign them to the atomic species, performing this task done quantitatively for a large number of frames quickly becomes a monumental feat.

#### Imaging in *k*-space

**Figure 12:**
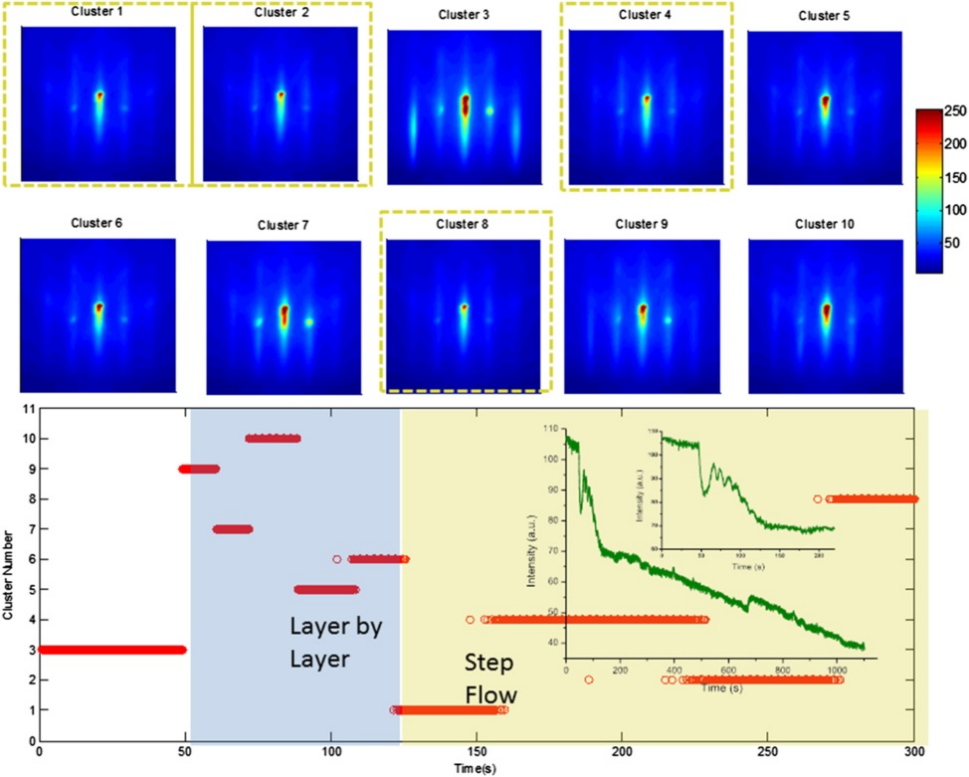
Clustering on RHEED image sequences. *K*-means analysis of the first segment of the RHEED movie (of SrRuO3 growing on (001) SrTiO_3_) was performed. The mean of all members within each cluster for the ten clusters is shown in the upper panel of the figure, and the temporal dependence of the clusters is shown in the lower panel. Inset, below: The mean of the specular diffraction spot, with an expanded view of the first 220 s of the growth.

The concept of image frame analysis can be further extended to image sequences, which are perfectly suited for analysis using the same multivariate statistical methods. As an example, we turn to an image sequence of reflection high-energy electron diffraction (RHEED) data acquired during deposition of SrRuO_3_ on (001) SrTiO_3_. The (00) or specular spot is closely monitored for signs of oscillations, which would indicate a layer-by-layer growth of the film on the substrate. As a first approximation, these oscillations arise due to a filling of incomplete layers (which reduces step density and therefore increases the intensity of the diffracted beam), until the layer is complete followed by more roughening as more material is deposited, with a corresponding decrease in intensity of the specular spot [81]. The process continues as the deposition proceeds, and the resulting profile of the specular spot intensity over time is periodic if the growth mode is layer-by-layer. The intensity of the specular spot over the course of the deposition is shown inset in the lower panel in Figure 12, plotted as an olive line. This graph shows that, after the start of the deposition, oscillations can only be observed up to *t* ~ 110 s (see expanded inset in graph), and afterward, no oscillations are observed, indicating a transition to a step-flow growth mode.

We studied the first 220 s of growth by using *k*-means clustering, with ten clusters, with the mean of the clusters plotted in the upper half of Figure 12, while the temporal dependence of each cluster is graphed in the lower panel. After the deposition begins (at *t* = 50 s), there are five distinct clusters that characterize the growth process before the transition to step-flow mode. These highlight the pathway for the transition - it appears that it occurs with the streaks gradually losing intensity over time until they are more spot-like (seen in cluster 6). Beyond *t* = 120 s, four clusters characterize the remaining *t* = 100 s of growth, and these are outlined with olive dash lines in the upper panel. Interestingly, there is little difference between these clusters (compared with the clusters in the layer-by-layer growth segment), and moreover, the similarity suggests little roughening effects in the grown film. We can therefore assign, unambiguously, that the layer-by-layer growth transitions to the step-flow mode when cluster 1 is active, i.e., at *t* = 120 s. Thus, the *k*-means clustering allows identification of growth mode transitions as well as the pathway through which this occurs in *k*-space, and furthermore allows identification of existence or absence of surface roughening. The method is equally applicable to detect 2D → 3D growth mode transitions [82], disordered → ordered transitions [83], strain relaxation [84], etc.

#### Supervised learning: domain shape recognition

Principal component analysis combined with neural networks can be used for the analysis of ferroelectric domain shapes, which provides insight into the highly non-trivial mechanism of ferroelectric domain switching, and potentially establishes a new paradigm for the information encoding based on the capture domain shape in the image.

**Figure 13:**
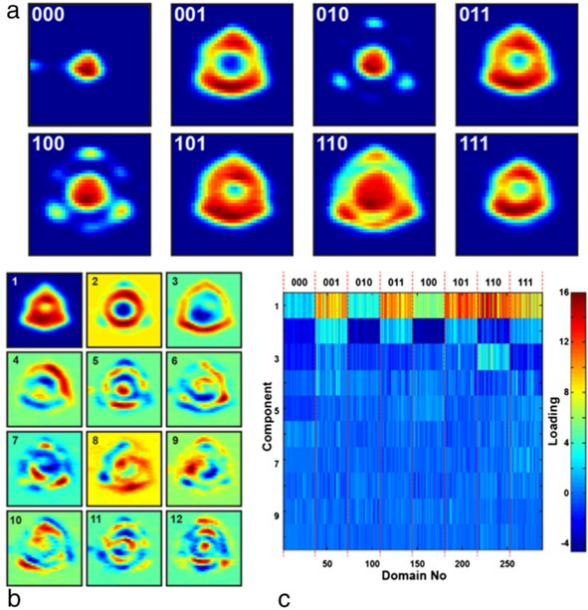
Recognition of the shape of the ferroelectric domains. **(a)** Shape of the ferroelectric domains produced by application of the sequences of positive and negative electric pulses to the SPM tip. **(b, c)** Principal component analysis over experimental data set of 288 domains. **(b)** First 16 eigenvectors and **(c)** weights.

Recent investigation of the SPM tip-induced ferroelectric domain switching by sequences of positive and negative electric pulses (labeled as a sequence of 0s and 1s) demonstrated unexpectedly complex, symmetrical, and asymmetrical morphologies of the formed domains (Figure 13a) [85]. These results suggest an intriguing possibility of practical applications in modern data storage devices, where data is encoded as a set of parameters that define domain shape and size. However, development of this approach into a viable device necessitates reliable analysis techniques which allow recognition of the sequence of the written electrical pulses via shape and size of the resulting domain. We illustrate a combinatorial PCA and neural network approach to address this problem [86].

The experimental data set consisted of PFM images of the domains produced by an application of a number of electrical pulses of varying length, and a total of 288 domains were acquired for testing.

We used PCA to obtain a set of the descriptors that characterized the individual domains. Each domain image consisted of *N* × *N* pixels and was unfolded into a 1D vector of *N*2 length. PCA eigenvectors (Figure 13b) and corresponding weight coefficients (Figure 13c) characterized the domain morphology. Color map of the weights demonstrates clear differences between the domain groups corresponding to different switching pulses (Figure 13c). This approach illustrates use of eigenvectors for characterization of all of the experimentally observed features of the domain morphology, and the weights can be used as an input parameter for the recognition by a feed-forward neural network.

For testing of this approach, the experimental data set was divided into training and test data sets. The PCA over the training data set (about 15% of the domains) was used for calculation of etalon eigenvectors, which was used for deconvolution of the testing weight coefficients over the test data set. The set of the training weights and corresponding switching sequences are then applied for neural network training. The set of testing weights are further used as inputs for recognition.

Experimental simulations of the suggested approach showed its practical applicability and demonstrated probability of the recognition above 65%; however, this relatively low value is mainly defined by irreproducibility of the switching process, caused by the non-ideal nature of the ferroelectric crystal.

### High-performance computing

**Figure 14:**
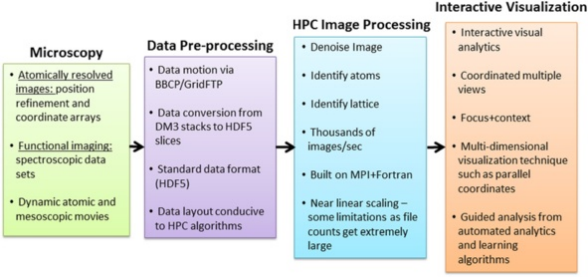
High-performance computation microscopy workflow. Life cycle of near-real-time analysis of large data from local imaging and spectroscopy experimental measurements.

The trend of the generated scientific data, by instruments, experiments, sensors, or supercomputer simulations, has historically been characterized by exponential growth, and this trend is anticipated to continue into the future [87,88]. As detailed in Table 1, the current scientific data volume output by local imaging and spectroscopy techniques is significant and will require high-performance computing platforms to meet the demands of analysis and visualization. There is clearly a need for a framework that will allow for near real-time processing and interactive visualization of scanning and electron microscopy data. Figure 14 exemplifies the hardware types and algorithms needed in the life cycle of scientific data, from the point of generation to analysis, visualization, and data archival. Customizable scalable methods, big data workflows, and visualization for scanning and electron microscopy data are detailed in the following sections.

#### Scalable methods

The key concepts in generating effective high-performance computing methods are managing latency of data transfer and balancing workload. Algorithms that are structured to effectively utilize the ever-increasing capacity of high-performance computing are called scalable methods. The movement of data in high-performance computing and across storage devices is well known, with hierarchies of transmission latency; therefore, analyzing scanning and electron microscopy data on these platforms will require physics-based algorithms that are customized to exploit parallel work while minimizing communication cost [89]. Experimental scientists will need to join with computational scientists and applied mathematicians to continue to scale this analysis to the next generation of high-performance computing (HPC) systems [90].

Future HPC systems are expected to have processor cores, memory units, communication nodes, and other components totaling in the hundreds of millions [91], and it is expected that faults in these systems will occur in the time frame of seconds [92]. This underscores the requirement of the algorithms specifically designed for analysis of scanning and electron microscopy data which must use robust workload balancing tools that are resilient to errors in algorithmic execution, as well as data transfer.

#### Big data workflows

To effectively leverage scalable methods for analysis on large-scale HPC systems, a sophisticated data workflow is required. Whereas computational scientists are accustomed to dealing with the idiosyncrasies of HPC environments (compiler technologies, scientific libraries, communication libraries, complex data models), microscopists generally are not. This presents a challenge in delivering the promise of near real-time analysis to the users at scanning probe, focused X-ray, tomography, and electron microscopy imaging facilities [89]. To overcome this challenge, we are employing an automated workflow-based approach.

In a typical example, the user will collect data from the instrument via the instrument control interface. As measurements progress, data is generated in a standard microscopy data format such as Digital Microscopy version 3 (DM3) or a text-based file (see Figure 14). Upon the completion of a measurement, the workflow begins, with the data transfer via a light communication node from the instrument to a high-performance storage [93]. This approach allows pipelining of the data to an HPC environment in parallel while subsequent measurements are taken at the instrument and other instruments are sending data.

Once the data file is stored within an HPC environment, the next stage of the workflow includes conversion to a data model suitable for HPC-based analysis, generally using the Hierarchical Data Format version 5 (HDF5). With the data set now converted and resident on a parallel file system, the next stage of the workflow, analysis via scalable methods, can be executed. At this juncture, an analysis algorithm is selected based on the instrument, the measurement, the material composition, and other user-specified criteria. Once selected, the analysis is executed on an HPC system. The resultant data and statistics are then made available to the user for inspection and further analysis. Initial experimentation of this concept has shown that analysis can be completed in seconds, allowing near real-time feedback from the measurement. Upon completion of the analysis, the data is then organized for possible archival. Once data movement and analysis is completed, interactive visual analysis is made available for further inspection of the data.

#### Scalable analytics

It is important to note that the difficulties surrounding scalable analytics in the context of the imaging methods insofar discussed extend far beyond the need for task-based and data-based parallelism. In particular, one of the primary challenges expected to impede further progress is the application of statistical methods in extremely high dimensions. Due to the structure of the analysis problems in computational settings, the complexity of the problem space manifests itself as a high-dimensional analysis problem, where dimensionality is most often associated with the number of measurements being considered simultaneously. The curse of dimensionality is a persistent phenomenon in modern statistics due to our ability to measure at rates and scales unheard of until the modern era [94]. However, there are many strategies to mitigate the statistical consequences of high dimensionality.

While some of the methods noted earlier in this paper are computationally scalable, in many cases, they are not appropriate for other reasons. For example, although PCA, ICA, *k*-means, and back propagation for neural networks all fit the Statistical Query Model, and thus belong to a known set of problems that can essentially scale linearly, this does not necessarily solve the issues raised by high-dimensional analysis [95]. For example, it is important to observe that in high-dimensional spaces, nearest neighbors become nearly equidistant [96]. This is particularly problematic for clustering algorithms but also has significant consequences for other dimensionality reduction techniques.

Clustering in high-dimensional spaces has been addressed using a variety of methods that consider scalability. A good example is the use of hashing in similarity measurements. Hashing techniques that facilitate neighborhood searches in high-dimensional space rely on various assumptions for tractability. Often, these assumptions include independence among the dimensions; in the case of Weiss et al., the authors suggest the use of PCA in order to prep the data in such a way that these assumptions are more accurate [97]. Moreover, various hashing techniques attempt to preserve distances between points in different ways, such that the user must be savvy enough to understand these assumptions in order to choose the best approach [97,98]. For example, Weiss et al. gains much of its power by only attempting to preserve the relative order of small distances. After a certain distance in space is reached, all distances beyond that are allowed to become equidistant in the space represented by the hash codes. However, this brings us back to the unfortunate situation that in extremely high dimensions, points tend to become equidistant, such that these hashing approaches cannot be expected to work for problems that do not have structure allowing some sort of dimensionality reduction.

We also suspect that many important patterns cannot be captured by linear dimensionality reduction techniques alone. However, non-linear techniques, such as those shown by Roweis et al., Tenenbaum et al., Belkin et al., and Gerber et al., are less scalable [99-102]. Many such methods fall under the umbrella of manifold learning, which is a technique meant to take advantage of cases where the data lie on a non-linear subspace that can be represented by a significantly smaller number of dimensions [103]. Many manifold learning approaches involve the solution of a symmetric diagonally dominant (SDD) linear system, but recent progress has been made in finding more efficient, scalable solutions to such problems [104].

When dimensionality reduction techniques still leave large numbers of potentially relevant measurements, other scalable approaches for dealing with high-dimensional analysis are still required. In the case of clustering, one such scalable approach that deals with high-dimensional clustering can be found in the methodology of Vatsavai et al. [105]. Note that this method also automatically attempts to select the number of clusters, a known problem for *k*-means clustering.

Many of the most effective solutions to the challenges presented by high-dimensional data have relied on the injection of additional knowledge. In the case where human expertise can play a part in pattern discovery and dimensionality reduction, data analysis becomes much easier. Unfortunately, more often than not, we are dealing with problems where the physics are unknown and the discovery of manual patterns is extremely difficult even in the case of deep domain knowledge. Thus, more automated methods for incorporating additional information, such as the integration of alternate imaging modalities, become important.

Moreover, methods of automated pattern discovery in large data sets have made great progress in recent years. In particular, in the case of imagery methods, much progress in automated feature extraction has occurred in the area known as deep learning [106]. However, such methods rely on large aggregated image repositories. This means that big data workflows have to be in place to retain large numbers of experimental results and allow their joint analysis. In addition, while these methods have proven to be scalable, they are also subject to finding many irrelevant patterns when utilizing networks consisting of extreme numbers of parameters [107].

#### Visualization

Dynamic hypothesis generation and confirmation techniques are a necessity for enabling scientific progress in extreme-scale science domains. Indeed, when insight is detected in the data, new questions arise, leading to more detailed examination of specific constituents. Accordingly, scientific analysis techniques should enhance the scientist’s cognitive workflow by intelligently blending human interaction and computational analytics at scale via interactive data visualization. The orchestration of human cognition and computational power is critical for two primary reasons: (i) the data are too large for purely visual methods and require assistance from data processing and mining algorithms, and (ii) the tasks are too exploratory for purely analytical methods and call for human involvement. Having established our strategy for harnessing computational power through automated analytical algorithms, we will devote the remainder of this section to several key strategies for integrating human-guided scientific analysis at scale in the materials sciences.

Given the scale and complexity of the materials data, a visual analytics approach is the most viable solution to accelerate knowledge discovery. Thomas et al. define visual analytics as ‘the science of analytical reasoning facilitated by interactive visual interfaces’ [108]. The fundamental goal of visual analytics is to turn the challenge of the information overload into an opportunity by visually representing information and allowing direct interaction to generate and test hypotheses. The advantage of visual analytics is that users can focus their full cognitive and perceptual capabilities on the analytical process, while simultaneously leveraging advanced computational capabilities to guide the discovery process [109]. Visual analytics is a modern take on the concept of exploratory data analysis (EDA) [110]. Introduced by Tukey, EDA is a data analysis philosophy that emphasizes the involvement of both visual and statistical understanding in the analysis process.

To allow efficient EDA in materials science, the combination of multiple views (CMV) and focus + context information visualizations are needed. CMV is an interaction methodology that involves linked view manipulations distributed across multiple visualizations, and recent evaluations demonstrate that this approach fosters more creative and efficient analysis than non-coordinated views [111]. In a CMV system, as the scientist manipulates a particular visualization (e.g., item selections, filtering, variable integrations), the manipulations are immediately propagated to the other visualizations using a linked data model. In conjunction with CMV, focus + context representations support efficient EDA by preserving the context of the more complete overview of the data during zooming and panning operations. As the scientist zooms into the data views to see more details, the focus + context display simultaneously maintains the context or gestalt [112] of the entire data set. In this way, the operator is less likely to lose their orientation within the overall data space while investigating fine-grain details.

**Figure 15:**
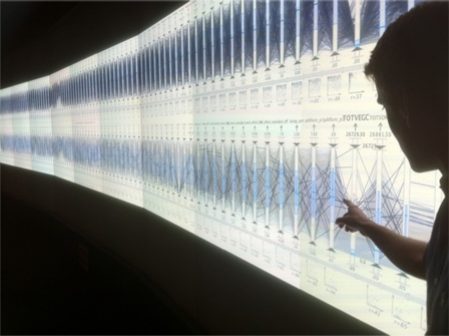
Lossless multidimensional visualization. EDEN is used to visually analyze a 1,000 simulation CLM4 point ensemble data set with 81 parameters and 7 output variables on ORNL’s EVEREST power wall facility which offers 115,203,072 (35 million) pixels. EDEN is a promising technique for materials science data analysis especially when it is coupled with dimensionality reduction and statistical learning algorithms.

Given the need to analyze multiple dimensions in materials science scenarios, multidimensional information visualization techniques that enable comparative studies are required. In conjunction with the dimensionality reduction techniques, previously mentioned, lossless multidimensional visualizations are also desired. A promising solution is to use an approach similar to the Exploratory Data Analysis Environment (EDEN) system [113], which is built around a highly interactive variant of the parallel coordinates technique. Inselberg initially popularized the parallel coordinates technique as an approach for representing hyper-dimensional geometries [114]. In general, the technique yields a compact two-dimensional representation of multidimensional data sets by representing the *N*-dimensional data tuple *C* with coordinates (*c*_*1*_, *c*_*2*_,…, *c*_*N*_) [115] on *N* parallel axes that are joined with a polyline. In theory, the number of dimensions that can be displayed is only limited by the horizontal resolution of the display devices (i.e., Figure 15 shows a particular parallel coordinates plot in EDEN that accommodates the simultaneous display of 88 variable axes). Consequently, parallel coordinates avoid the loss of information afforded by dimensionality reduction techniques. But in a practical sense, the axes that are immediately adjacent to one another yield the most obvious information about relationships between attributes. In order to analyze attributes that are separated by one or more axes, interactions and graphical indicators are required. Several innovative extensions that seek to improve interaction and cognition with parallel coordinates have been described in the visualization research literature. For example, Hauser et al. [116] described a histogram display, dynamic axis re-ordering, axis inversion, and details-on-demand capabilities for parallel coordinates. The literature covering parallel coordinates is vast and covers multiple domains as recently surveyed by Heinrich and Weiskopf [117].

EDEN extends the classical parallel coordinates axis by providing cues that guide and refine the analyst’s exploration of the information space. This approach is akin to the concept of the scented widget described by Willett et al. [118]. Scented widgets are graphical user interface components that are augmented with an embedded visualization to enable efficient navigation in the information space of the data items. The concept arises from the information foraging theory described by Pirolli and Card [119], which relates human information gathering to the food foraging activities of animals. In this model, the concept of information scent is identified as the ‘user perception of the value, cost, or access path of information sources obtained by proximal cues’ [119]. In EDEN, the scented axis widgets are augmented with information from automated data mining processes (e.g., statistical filters, automatic axis arrangements, regression mining, correlation mining, and subset selection capabilities) that highlight potentially relevant associations and reduce knowledge discovery timelines.

The parallel coordinates plot is ideal for exploratory analysis of materials science data because it accommodates the simultaneous display of a large number of variables in a two-dimensional representation. In EDEN, the parallel coordinates plot is extended with a number of capabilities that facilitate exploratory data analysis and guide the scientist to the most significant relationships in the data. A full description of these extensions is beyond the scope of this article, but the reader is encouraged to explore prior publication for more detailed explanations of our multidimensional analysis techniques [113,120]. EDEN is an exemplary case of the indispensable visual analytics techniques that provide intelligent user interfaces by leveraging both visual representations and human interaction, thereby enhancing scientific discovery with vital assistance from automated analytics. As we develop new visual analytics approaches like EDEN for materials science workflows, we expect to dramatically reduce knowledge discovery timelines through more intuitive and exploratory analysis guided by machine learning algorithms in an intelligent visual interface.

## Conclusions

The development of electron and scanning probe microscopies in the second half of the twentieth century was enabled by computer-assisted methods for automatic data acquisition, storage, analysis, and tuning and refinement of feedback loops as well as imaging parameters. In the last decade, high-resolution STEM and STM imaging techniques have enabled acquisition of high-veracity information [121] at the atomic scale, readily providing insight on positions and functionality of materials that have been inaccessible due to a lagging analysis framework in the microscopy communities. Naturally, progress in complexity of dynamic and functional imaging leads to multidimensional data sets containing spectral information on local physical and chemical functionalities, which can be easily expanded further to acquire data as a function of a plethora of parameters such as time, temperature, or many other external stimuli.

Maximizing the scientific output from existing and future microscopes brings forth the challenge of analysis, visualization, and storage of data, as well as decorrelation and classification of the known and unknown hidden data parameters, the traditional big data analysis. The existing infrastructure for such analysis has been developed in the context of medical and satellite imaging, and its extension to functional and structural imaging data is a natural next step. Of course, further development toward a flexible infrastructure where the scientists can select or define their own analysis algorithms to analyze the data ‘on the fly’ as it is being collected can be envisioned. This will require scalable algorithms, high-performance computing, and storage infrastructure. Reducing the data sets to a more manageable size, while initially attractive, comes with the risk of losing significant information within the data, particularly for exploratory studies in which the phenomena of interest may not be captured by statistical methods.

Beyond the big data challenges [122,123] is the transition to a deep data approach, in which we fully utilize all the information present within the data to derive an understanding [124] - namely, how do we ascribe relevant physical and chemical information contained within the data sets, differentiate relevant and coincidental behaviors, move beyond simple correlation, and link to scientific theory? High-resolution imaging allows us to explore the microscopic degrees of freedom in the system - how can we use theory to understand these behaviors, refine theoretical models, and ultimately enable knowledge-driven design and optimization of new materials? [125]. To achieve this goal, new methods and theories will be necessary for defining the local chemical and physical descriptions, their spatial distribution and evolution during reactions. While complicated, recent progress in information and statistical theory suggest that such descriptions are possible [126].

One of the approaches to achieve this goal is through the user center model that combines development and maintenance of cutting edge tools, as well as experience and detailed knowledge of data interpretation in terms of relevant behaviors, all while maintaining an open access policy - making the findings available to the broader scientific community. Equally important will be the cross-disciplinary synergy between theory, imaging, and data analytics, harnessing the power of multivariate statistical methods to understand and explore multidimensional imaging and spectroscopy data sets.

Integration of the knowledge in the field will allow development of universal database libraries allowing identification and data mining of novel and well-understood materials, refinement and improvement of dynamic data, and ultimately creation of supervised expert systems that will allow rapid identification and analysis of unknown systems. Successes in fields such as medical diagnostics and imaging suggest that this is fully possible. These developments will further open the pathway for exploration and tailoring of desired material functionalities based on better information. We anticipate the emergence of Google-like environments that will allow storage and interpretation of collective knowledge and image interpretation in the context of data and historical knowledge. Rather than creating multiple samples, the structure-property relationships extracted from a single disordered sample could offer a statistical picture of materials functionality, providing the experimental counterpart to Materials Genome-type programs.

## Abbreviations

AC: alternating current
AFM: atomic force microscopy
ANN: artificial neural network
BE: band excitation
BEPS: band excitation polarization spectroscopy
c-AFM: conductive atomic force microscopy
CFO-BFO: CoFe_2_O_4_-BiFeO_3_
CITS: current imaging tunneling spectroscopy
CMV: combination of multiple views
DC: direct current
DM3: Digital Microscopy version 3
EDA: exploratory data analysis
EDEN: Exploratory Data Analysis Environment
FFT: fast Fourier transfrorm
FORC: first-order reversal curve
FORC-IV: first-order reversal curve current-voltage
HPC: high-performance computing
HDF5: Hierarchical Data Format version 5
ICA: independent component analysis
IV: current-voltage
PCA: principal component analysis
PFM: piezoresponse force microscopy
RHEED: reflection high-energy electron diffraction
SPM: scanning probe microscopy
SS PFM: switching spectroscopy piezoresponse force microscopy
STEM: scanning transmission electron microscopy
STEM-HAADF: scanning transmission electron microscopy high-angle annular dark-field imaging
STM: scanning tunneling microscopy
STS: scanning tunneling spectroscopy
SSD: symmetric diagonally dominant
*ω*: frequency
